# Ayurveda in Knee Osteoarthritis—Secondary Analyses of a Randomized Controlled Trial

**DOI:** 10.3390/jcm11113047

**Published:** 2022-05-28

**Authors:** Christian S. Kessler, Michael Jeitler, Kartar S. Dhiman, Abhimanyu Kumar, Thomas Ostermann, Shivenarain Gupta, Antonio Morandi, Martin Mittwede, Elmar Stapelfeldt, Michaela Spoo, Katja Icke, Andreas Michalsen, Claudia M. Witt, Manfred B. Wischnewsky

**Affiliations:** 1 Institute of Social Medicine, Epidemiology and Health Economics, Charité—Universitätsmedizin Berlin, Corporate Member of Freie Universität Berlin and Humboldt-Universität zu Berlin, 10117 Berlin, Germany; michael.jeitler@charite.de (M.J.); katja.icke@charite.de (K.I.); andreas.michalsen@charite.de (A.M.); claudia.witt@charite.de (C.M.W.); 2 Department for Complementary and Integrative Medicine, Immanuel Hospital Berlin, 14109 Berlin, Germany; elmar.stapelfeldt@immanuelalbertinen.de (E.S.); michaelaspoo@web.de (M.S.); 3 Faculty of Ayurveda, Institute of Medical Sciences, Banaras Hindu University, Varanasi 221005, India; dr_ks_dhiman@yahoo.co.in; 4 Dr. Sarvepalli Radhakrishnan Rajasthan Ayurved University, Jodhpur 342037, India; ak_ayu@yahoo.co.in; 5 Department of Psychology and Psychotherapy, University of Witten Herdecke, 58455 Witten, Germany; thomas.ostermann@uni-wh.de; 6 European Academy of Ayurveda, 95018 Birstein, Germany; guptayurveda@yahoo.com (S.G.); martin.mittwede@ayurveda-akademie.org (M.M.); 7 Department of Kaya Cikitsa, J.S. Ayurveda College & P.D. Patel Ayurveda Hospital, Nadiad 387001, India; 8 Ayurvedic Point, School of Ayurvedic Medicine, 20149 Milan, Italy; dr.morandi@ayurvedicpoint.it; 9 Department of Religious Sciences, University of Frankfurt, 60323 Frankfurt, Germany; 10 Institute for Complementary and Integrative Medicine, University Hospital and University of Zurich, 8091 Zurich, Switzerland; 11 Center for Integrative Medicine, University of Maryland School of Medicine, Baltimore, MD 21201, USA; 12 Department of Mathematics and Computer Science, University of Bremen, 28359 Bremen, Germany; wischnewsky@escience.uni-bremen.de

**Keywords:** Ayurveda, traditional Indian medicine, knee osteoarthritis, complementary medicine, integrative medicine

## Abstract

Background: Ayurveda is widely practiced in South Asia in the treatment of osteoarthritis (OA). The aim of these secondary data analyses were to identify the most relevant variables for treatment response and group differences between Ayurvedic therapy compared to conventional therapy in knee OA patients. Methods: A total of 151 patients (Ayurveda *n* = 77, conventional care *n* = 74) were analyzed according to the intention-to-treat principle in a randomized controlled trial. Different statistical approaches including generalized linear models, a radial basis function (RBF) network, exhausted CHAID, classification and regression trees (CART), and C5.0 with adaptive boosting were applied. Results: The RBF network implicated that the therapy arm and the baseline values of the WOMAC Index subscales might be the most important variables for the significant between-group differences of the WOMAC Index from baseline to 12 weeks in favor of Ayurveda. The intake of nutritional supplements in the Ayurveda group did not seem to be a significant factor in changes in the WOMAC Index. Ayurveda patients with functional limitations > 60 points and pain > 25 points at baseline showed the greatest improvements in the WOMAC Index from baseline to 12 weeks (mean value 107.8 ± 27.4). A C5.0 model with nine predictors had a predictive accuracy of 89.4% for a change in the WOMAC Index after 12 weeks > 10. With adaptive boosting, the accuracy rose to 98%. Conclusions: These secondary analyses suggested that therapeutic effects cannot be explained by the therapies themselves alone, although they were the most important factors in the applied models.

## 1. Introduction

Ayurveda is by far the most relevant traditional medical system (TMS) in South Asia, and represents a TMS of major social penetration in its countries of origin. In recent decades, Ayurveda has also become increasingly popular in Western countries [1,2,3,4].

Since 9 November 2014, Ayurveda has been regulated by an independent ministry in India (Ministry of Ayurveda, Yoga, Naturopathy, Unani, Siddha, Sowa-Rigpa and Homoeopathy; abbreviated as AYUSH Ministry) to ensure its optimal development and dissemination in medical practice, both inside and outside of India [5]. Professional guidelines for Ayurveda education, research, pharmacovigilance, and practice have been and continue to be issued in India [4,5,6,7,8]. According to the World Health Organization (WHO), Ayurvedic therapies can represent effective treatment options for certain diseases [9]. Outside of South Asia, the political, scientific, and professional medical recognition of Ayurveda is still in a pioneer stage [10,11,12,13,14].

In Western contexts, Ayurveda is predominantly used within the framework of complementary and integrative medicine (CIM) and wellness [15]. In recent years, its application has increasingly fallen within the scope of integrative medicine (IM) practice in Western countries, an approach that aims to explicitly combine conventional medicine and complementary interventions in a holistic, patient-centered, evidence-informed manner [16,17,18,19]. The WHO considers traditional Indian medicine (TIM) interventions to be medically and economically beneficial, especially as a treatment option for chronic diseases [20,21]. In February 2022, new benchmarks for training and practice in Ayurveda were published by the WHO [20,21].

On a global scale, the research base for Ayurveda according to evidence-based medicine (EbM) is still weak. Despite numerous initiatives and announcements to improve the scientific visibility of Ayurveda (especially in South Asian countries), the number of internationally published, high-quality publications is still disproportionately low in comparison to other CIM and TMS treatment approaches, such as yoga, acupuncture or mind-body medicine (MBM), or other whole systems of medicine (WMS) such as traditional Chinese medicine (TCM) [22].

Areas of exception to this are musculoskeletal disorders and pain syndromes, diagnoses that notably figure prominently in Ayurvedic treatment contexts; for example osteoarthritis (OA), rheumatoid arthritis (RA), back pain, and fibromyalgia [3,23,24,25,26,27,28]. Considerable clinical evidence of methodologically sound studies, some of them exploratory and some of them confirmatory, is already available, and predominantly suggests clinically relevant effectiveness.

The main results of this clinical trial—the first larger confirmatory randomized controlled trial (RCT) on the effectiveness of Ayurveda on patients with osteoarthritis of the knee (OA)—suggested that a traditional Ayurveda therapy might be superior to conventional standard therapy when considering all potentially confounding factors [3]. Furthermore, treatment effects were sustained at the 12-months long-term follow-up [3]. Observations and analyses from a nested diagnostic study (*n* = 30) provided additional valuable data regarding intra- and interrater reliability in this specific diagnostic context, and they have also been published elsewhere [26]. Overall, methodology and results have been published in three previous publications [3,26,27] and in a preceding systematic review [29].

Nevertheless, several open questions in this area; for example, ones related to specific modes of action, mechanisms, multimodality treatments and the significance of unspecific/setting/placebo effects, remain largely unanswered regarding the effectiveness of Ayurveda (as pars per toto for WMS and TMS) [3,26,27,29].

For this particular reason, data from the parent RCT were further analyzed in detail in order to illuminate and thus better understand where exactly Ayurveda—using the concrete example of Ayurvedic OA treatment—might be effective, and which particular clinical variables are interrelated to each other and in what way [3,26,27].

## 2. Materials and Methods

### 2.1. Methodology of the Parent RCT

Details of the RTC’s study protocol, a related systematic review, the main findings of this RCT, and the results of an embedded diagnostic reliability study in this international research collaboration project have already been published elsewhere [3,26,27,29].

In summary, 151 patients with knee OA were included in this confirmatory, multicenter, randomized, controlled clinical trial and treated in two hospital departments and two outpatient practice settings in Germany [3]. Following random group allocation, patients received either multimodal traditional Ayurvedic treatment or guideline-based multimodal conventional conservative treatment. The interventions are described in detail in the main publication [3]. Each patient received 15 individual treatments for 12 weeks; the total observation period per patient, including follow-up visits, was one year. The primary endpoint of the study was the change at 12 weeks in the WOMAC Index in the between-group comparison. The WOMAC Index inventory precisely assesses the diagnosis-specific health status of knee and hip osteoarthritis patients, has been translated to several languages, and is used worldwide in clinical osteoarthritis research [30]. Secondary outcomes included the WOMAC Index subscales, pain questionnaires, numerical rating scales on pain and sleep quality, quality of life and psychometric questionnaires, use of rescue medication, and safety aspects. No secondary analyses had previously been performed and published.

### 2.2. Statistics

For the main analysis of the RCT, an intention-to-treat analysis was performed involving all 151 randomized study participants [3]. The primary outcome was the change in the WOMAC Index after 12 weeks [31,32]. The sum of the WOMAC Index in the validated German version ranges from 0 (no pain, no stiffness, no functional limitations in everyday life) to 240 (extreme pain, extreme stiffness, and extreme functional limitations in everyday life) [32].

For these secondary analyses, missing data for the 151 patients were replaced using multiple imputation by applying an iterative Markov chain Monte Carlo (MCMC) method. Prior to imputing data, an exploratory analysis to assess MCMC convergence was run. Using the SPSS output management system (OMS) utility, the 20 imputed datasets were compressed into a final single dataset.

Nominal values were described by their absolute and relative frequencies. Quantitative values were characterized by means and their standard deviations or ranges (maximum–minimum). The Wilcoxon–Mann–Whitney test, as a parameter-free statistical test, was used to test the significance of the agreement of distributions. The Kolmogorov–Smirnov test was used to examine the normal distributions of the WOMAC Index at different time points.

Univariate generalized linear models (GLMs) were used to reduce within-group error variance and remove confounding factors. To test the assumptions for the GLMs, Levene’s test for homogeneity of variances was performed. In the GLMs, treatment (Ayurveda, conventional therapy) and gender were each included as fixed factors, the study center as a random factor, and the WOMAC Index baseline as covariate. In addition, patient expectations of their therapy received and the role of nutritional supplements for patients with Ayurveda were also examined. The “estimated marginal means” (=estimates) of the WOMAC Index after 12 weeks resp. 12 months and all possible interactions were calculated and compared. In addition, model goodness and effect sizes (partial *η^2^* and Cohen’s d) were calculated for each factor and covariate.

Tree decision algorithms (chi-squared automatic interaction detector (Exhausted CHAID), classification and regression trees (CARTs), and entropy-based decision trees (C5.0)) were used to detect relationships between groups and predict future events. To improve the quality of the C5.0 model, AdaBoost (short for Adaptive Boosting), a statistical classification meta-algorithm, was used [33].

In addition, a normalized Gaussian radial basis function (normalized RBF) network was used as another predictive model. RBF networks are a commonly used type of artificial neural network for function approximation problems. An RBF network is a feedforward, supervised learning network with an input layer, a hidden layer called the radial basis function layer, and an output layer. The hidden layer transforms the input vectors into radial basis functions. The goal of an RBF network is to approximate the target function through a linear combination of radial kernels. The importance of an independent variable in the RBF network is a measure of how much the network’s model-predicted value changes for different values of the independent variable. Normalized importance is simply the importance value divided by the largest importance value, and is expressed as percentages.

Model-specific and model-agnostic methods for assessment of variable importance were applied. Examining the importance of an explanatory variable has several purposes.

- Model simplification: variables that are not important; i.e., that do not influence a model’s predictions, may be excluded from the model.
- Domain-knowledge-based model validation: identification of the most important variables may be helpful in assessing the validity of the model based on domain knowledge.
- Model exploration: comparison of variables’ importance in different models may help in discovering interrelations between the variables.

The analysis was performed using R version 4.1.2 (R Foundation, Vienna, Austria) combined with IBM SPSS Statistics version 28.01 (IBM Corp., Armonk, NY, USA).

## 3. Results

In the RCT, 151 patients with knee OA from two study centers were included (Ayurveda group: *n* = 77; conventional group: *n* = 74) [3]. Patients had a mean age of 61.2 ± 6.6 years (min 41; max 70). A total of 92.7% of the patients had concomitant diagnoses (Ayurveda 92.2%; conventional therapy 93.2%) and a WOMAC Index of 92.6 ± 42.2 (Ayurveda 91.1 40.3, maximum 162; conventional therapy 94.2 ± 44.4, maximum 188) at baseline (Table S1 in the Supplementary Materials) [3]. The average number of treatment sessions was 13.5 ± 1.7 for Ayurveda participants and 14.0 ± 2.7 for conventional participants. Mean treatment duration time was 67.8 ± 4.1 min (90.2 ± 5.8 min in the Ayurveda group and 45.3 ± 2.5 min in the conventional group) [3].

In the conventional group, the mean WOMAC Index improved from 94.2 ± 44.4 at baseline to 62.2 ± 43.1 after 12 weeks (*p* < 0.001), and in the Ayurveda group, from 91.1 ± 40.3 to 30.0 ± 26.9 (*p* < 0.001). Both interventions improved the WOMAC Index significantly (*p* < 0.001) and the bias-corrected standardized effect sizes (Cohen’s d (95% CI)) for the conventional group: 0.73 (0.39; 1.06); and for the Ayurveda group: 1.77 (1.40; 2.15).

**Figure 1:**
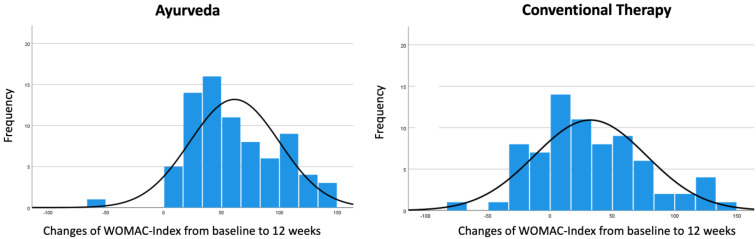
Frequency distribution of the changes in the WOMAC Index from baseline to 12 weeks with normal curve. Values > 0 of changes in the WOMAC Index indicate improvements, while values < 0 indicate worsening.

The frequency distributions of the changes in the WOMAC Index from baseline to 12 weeks for patients in the Ayurveda group and the conventional group are shown in Figure 1.

**Figure 2:**
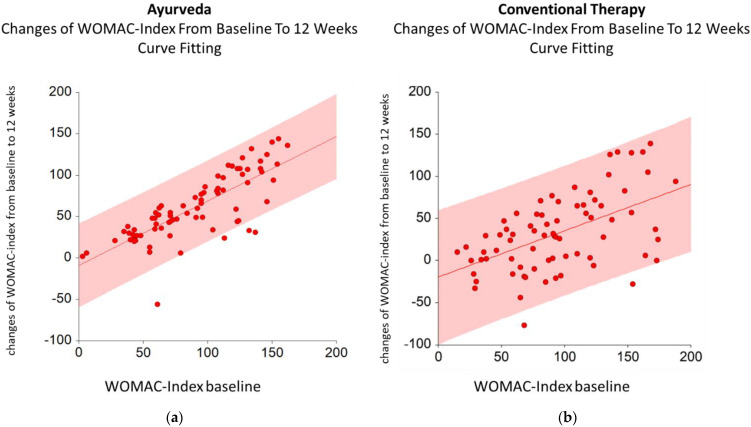
Estimated models for Ayurveda (**a**) and conventional therapy (**b**) with 95% CI for changes in the WOMAC Index from baseline to 12 weeks and WOMAC Index baseline.

The changes in the WOMAC Index depended linearly on the WOMAC Index baseline. The estimated linear regression model for Ayurveda is given by the following formula: changes in WOMAC Index from baseline to 12 weeks = A + Bx (WOMAC Index baseline), with A = −9.0 and B = 0.8 (95% CI for A (−23.1; 5.1) and 95% CI for B (0.6; 0.9)) (Figure 2a). The estimated linear regression model for conventional therapy is given by changes in the WOMAC Index from baseline to 12 weeks = A + Bx (WOMAC Index baseline), with A = −19.6 and B = 0.5 (95% CI for A (−41.0; 1.7) and 95% CI for B (0.3; 0.8)) (Figure 2b).

Next, the estimated marginal means (=estimates) of the changes in the WOMAC Index from baseline to 12 weeks resp. 12 months stratified by therapy, patients’ gender, and patients’ expectations of the therapy received as fixed factors; and WOMAC-A (pain), WOMAC-B (stiffness), and WOMAC-C (functional limitations) at baseline as covariates, as well as all possible interactions, were calculated and compared.

The estimates of the changes in the WOMAC Index from baseline to 12 weeks were 46.7; std. error (SE) 6.7 (95% CI 33.5–59.9) for Ayurveda; and 26.4; SE 6.7 (95% CI 13.2–39.7) for conventional therapy (Table 1). The between-group difference of 20.3 (95% CI 1.5–39.0) at 12 weeks was significant (*p* = 0.034) (Table 1). The estimates of the changes in the WOMAC Index from baseline to 12 months were 34.7; SE 7.0 (95% CI 20.8–48.6) for Ayurveda; and 25.4; SE 7.6 (95% CI 10.3–40.6) for conventional therapy (Table 1). The between-group difference of 9.3 (95% CI −29.8–10.3) at 12 months was no longer significant (*p* = 0.375) (Table 1).

The estimated marginal means of changes in the WOMAC Index from baseline to 12 weeks were 46.7 (SE 6.7) for Ayurveda and 26.4 (SE 6.7) for the conventional therapy, with therapy, gender, and patients’ expectations for therapy received as fixed factors and WOMAC-A (pain), WOMAC-B (stiffness), and WOMAC-C (functional limitations) at baseline as covariates, as well as all possible interactions (Table 1). The difference between Ayurveda and conventional therapy was 20.3 (SE 9.5).

Tests of between-subject effects showed that the effect size of the corrected model with the dependent variable “changes of WOMAC-Index from baseline to 12 weeks” was large (partial eta squared = 0.54); *p* < 0.001; observed power = 100%). Therapy (*p* = 0.007) and WOMAC-C (functional limitations; *p* = 0.001) were the only significant parameters in this model (Table 2). WOMAC-A (pain) (*p* = 0.196), WOMAC-B (stiffness) (*p* = 0.112), gender (*p* = 0.394), expectations for therapy received (*p* = 0.242), as well as interactions, were not significant. After 12 months, functional limitations at baseline (WOMAC-C) was the only significant parameter (*p* = 0.016).

In addition, if we added in a standard linear model (with forward stepwise as the model selection mode and the information criteria (AICC) as criteria for entry/removal) using BMI, age, duration of pain (years), and study center, then therapy and functional limitations at baseline were the most important predictors (Figure S1 in the Supplementary Materials). The function predicted by the observed is given by the following equation: Predicted value = 26.38 + 0.49x (Change in WOMAC Index from baseline to 12 weeks).

### 3.1. Role of Nutritional Supplements in the Primary Outcome in the Ayurveda Group

In the Ayurveda group with 77 patients, a total of 67.5% (n = 52) received nutritional supplements, whereas in the conventional group, none of patients received nutritional supplements. So, we examined the role of nutritional supplements in the change in the WOMAC Index from baseline to 12 weeks in the Ayurveda group. Of these 77 Ayurveda participants, 85.2% in study center 1 and 0% in study center 2 received nutritional supplements. Therefore, we included the study center as a random factor in a GLM model, and also considered interactions between all variables.

The estimate of the changes in the WOMAC Index from baseline to 12 weeks for Ayurvedic patients with supplements was 52.6; SE 3.4 (95% CI 45.7–59.4); and 52.2; SE 4.2 (95% CI 43.9–60.5) for patients without nutritional supplements. The difference of 0.4; SE 3.7 between these two subgroups was not significant (*p* = 0.920). Covariates appearing in the model were evaluated at the following values: WOMAC-A (pain) at baseline = 18.6; WOMAC-B (stiffness) at baseline = 9.8; WOMAC-C (functional limitations) at baseline = 61.8.

### 3.2. Importance of Multiple Independent Variables in the Primary Outcome

The importance of variables measures how much a model’s performance changes if the effect of a selected explanatory variable, or of a group of variables, is removed. The larger the change in the performance, the more important the variable. If different models are used, often different results will be obtained due to stochastic learning algorithms or of stochastic evaluation procedures.

Next, we used a radial basis function network to calculate the importance of multiple/different influencing factors in the change in the WOMAC Index from baseline to 12 weeks.

**Figure 3:**
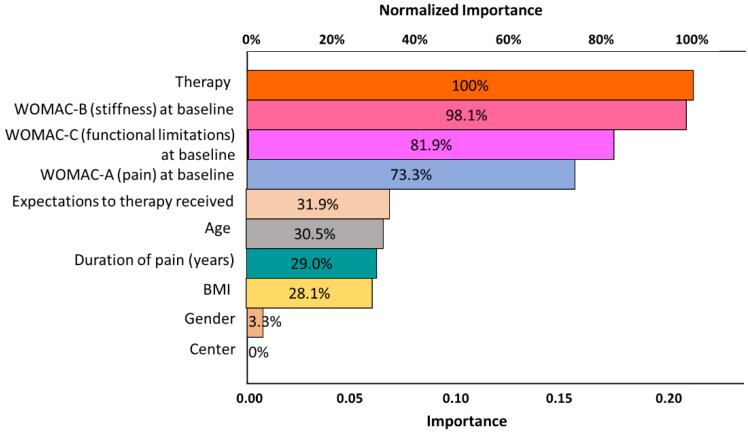
Results of the radial basis function network showing importance and normalized importance of various independent variables for the dependent-variable changes in the WOMAC Index from baseline to 12 weeks.

The results are shown in Figure 3. Therapy and the baseline WOMAC Index (WOMAC-A, WOMAC-B, and WOMAC-C) were the most important outcome-relevant independent factors, followed by patients’ expectations for therapy received, age, duration of pains (years), and BMI.

### 3.3. Significance of the WOMAC Index Subscales in the Therapeutic Effect

When examining the changes in the WOMAC Index from baseline to 12 weeks and its dependence on the single WOMAC subscales (pain, stiffness, and functional limitation) before the onset of therapy (baseline) for all patients, it was found that the outcome depended linearly on each individual subscale. The higher the value of a subscale baseline, the greater the improvement in the WOMAC Index after 12 weeks, independent of the therapy. For the WOMAC subscale of functional limitation (WOMAC-C) baseline, we obtained the following estimated linear equations:

- Ayurveda: changes in WOMAC Index from baseline to 12 weeks = −2.30 + 1.03x (WOMAC-C baseline);
- Conventional therapy: changes in WOMAC Index from baseline to 12 weeks = −14.46 + 0.72x (WOMAC-C baseline).

The decision tree (Figure S2 in the Supplementary Materials) for mean changes in the WOMAC Index from baseline to 12 weeks depending on the WOMAC-C baseline and therapy (cp. Table 3, model IV) split the WOMAC-C into four subgroups, with highly significantly (*p* < 0.001) different mean changes in the WOMAC Index from baseline to 12 weeks. The four subgroups were defined by the cutoff values of 50, 70, and 90. For instance, if WOMAC-C was in the interval (70, 90) (i.e., WOMAC-C > 70 and ≤90), then the mean change in the WOMAC Index after 12 weeks was 65.6 ± 36.3 (conventional therapy: 43.0 ± 33.5; Ayurveda: 79.9 ± 30.9).

In a CART model, Ayurveda patients with functional limitations (WOMAC-C) >60 at baseline and pain (WOMAC-A) > 5 had the greatest improvements in the WOMAC Index from baseline to 12 weeks, with a mean value 107.8 ± 27.4. On the other hand, the lowest mean value for the changes in the WOMAC Index from baseline to 12 weeks had Ayurveda patients with functional limitations (WOMAC-C) ≤60 at baseline and pain (WOMAC-A) ≤5.5. The mean value for these patients was 18.1 ± 26.2. Patients with conventional therapy and functional limitations (WOMAC-C) >65 at baseline had the greatest improvements in the WOMAC Index from baseline to 12 weeks, with a mean value 58.7 ± 44.8. If the functional limitations (WOMAC-C) were ≤65 at baseline, then the mean value for the changes in the WOMAC Index from baseline to 12 weeks was only 11.1 ± 32.6 WOMAC points.

Finally, when comparing various general linear models (Table 3), we found, as already previously stated, that therapy and functional limitations were the only significant predictors for the change in the WOMAC Index from baseline to 12 weeks in all models with WOMAC subscales. Both variables had a power >90% in all of the univariate general linear models, whereas WOMAC-A and WOMAC-B were underpowered in models V and VI. Based on the fact that therapy as a fixed factor was significant, we concluded that not all the level means were equal. Based on the fact that the covariate functional limitations at baseline were significant, we concluded that changes in the value of the covariate were associated with changes in the mean response value. Furthermore, these models showed that the significance of a variable could vary from model to model (WOMAC-B (stiffness)).

### 3.4. Prediction of Clinical Improvement

Decision trees (DTs) are a nonparametric supervised learning method used for classification and regression. The goal was to create a decision tree resp. equivalently to a set of rules that predicted the “change of WOMAC-Index from baseline to 12 weeks” respective to clinical improvement by learning simple decision rules inferred from the data features. The minimal important difference (MID) is an important characteristic of this patient-reported outcome measure (PROM). The minimal important difference (MID) or minimal clinically important difference (MCID) was defined as the “smallest change in a treatment outcome that an individual patient would identify as important and which would indicate a change in the patient’s management”. In Ref. [34], in line with the assumption in the main publication, we took a difference of 10 points for MID [3] as well as with the results in [34]. Next, we aimed at investigating which of the patients with Ayurveda or conventional therapy predictably showed a clinical improvement based on data at the beginning of the therapy. A total of 89.4% of the patients were correctly classified by the following ruleset (Table S2 in the Supplementary Materials), and with adaptive boosting [33], even 98%. 118 out of 119 patients with clinical improvement were correctly classified. In addition, 75 out of 77 Ayurveda patients were covered by rule 1; i.e., Ayurveda patients with a WOMAC functional limitations baseline >6 showed clinical improvement with a confidence of 94.8%. Only three of these patients did not belong to the class predicted by rule 1.

If we also used adaptive boosting [33] with 10 classifiers, then 148 out of 151 patients (98%) were correctly classified. In particular, all 119 patients with clinical improvement were correctly classified. None of the above nine predictors could be omitted in these classifiers without losing outcome quality.

## 4. Discussion

In this publication, data from an RCT on Ayurveda in patients with knee OA were further statistically analyzed using different statistical approaches [3,27]. In these secondary analyses, the therapy arms and the subscales of the WOMAC Index at baseline were the most important variables in the significant between-group differences in the WOMAC Index from baseline to 12 weeks in favor of Ayurveda. The intake of nutritional supplements in the Ayurveda group did not seem to be a significant factor in changes in the WOMAC Index. Ayurveda participants with functional limitations >60 points and pain >25 points at baseline had the greatest improvements in the WOMAC Index from baseline to 12 weeks. Moreover, an entropy-based C5.0 model with boosting showed a very high predictive accuracy for the changes in the WOMAC Index from baseline to 12 weeks.

In this in-depth reanalysis, it was shown that in both groups, significant improvements in the main outcome parameter (WOMAC Index after 12 weeks) occurred.

The different computational models indicated that different factors were relevant in the development of the main outcome parameter. Notably, although the therapy had a significant statistical influence on the main outcome parameter, in all models, this was only one (but the main) factor among several factors that determined its variation. The fact that the WOMAC Index baseline value; i.e., the complaint intensity before the onset of therapy, was a significant factor in the changes in the WOMAC Index study course was rather less surprising—the higher the functional limitation before the start of therapy, the higher the probability that patients would benefit more intensely from therapy; this was especially true for the Ayurveda group. In addition, the observation that patients’ expectations were part of these models may not really be surprising against the background of nonspecific effects (for example, context effects, setting effects, and placebo and nocebo effects) that have already been discussed in other contexts [35,36]. However, it showed again that expectations for clinical trials are in general a variable that should not be underestimated in the measured outcomes [35,37]. It was noteworthy that in this study, both study centers and the gender of the study participants were not significant variables that influenced the outcome.

Another major finding of these secondary analyses was that the WOMAC subscale functional limitation, even when considering various other factors, was the most important variable in terms of the change in the primary outcome of the WOMAC Index at 12 weeks. In both groups, regarding the group difference, but especially within the Ayurveda group, the therapy thus seemed to have a particularly beneficial effect on the functional aspects of knee OA patient complaints. In the context of future studies in this area, this aspect should be given special consideration, both in the planning of the clinical intervention and in the careful selection of outcomes.

It was noteworthy that the intake of nutritional supplements in the Ayurveda group did not seem to be a significant factor in the WOMAC Index in this study arm. This calculation was only possible at all because, due to changes in the study protocol required by regulations of the ethics committee during the study, some of study participants no longer received nutritional supplements [3]. A difference could have been assumed here. The results were certainly in contrast to the clinical experience of many Ayurveda practitioners in South Asia, and warrant further studies related to these specific aspects of traditional multimodality approaches. The subgroup analyses did not reveal any relevant differences between Ayurveda patients who received nutritional supplements and those who did not regarding the primary outcome. As a limiting factor, it must be added that the number of study participants for this question was too small to derive any definite conclusions, and can at most provide hints for future discussions regarding the importance of nutritional supplements in this context. In addition, future pharmacological studies in this area should be developed from a more TMS-contextualized perspective [24].

Compared to other CIM interventions, our study interventions have shown similar (and in part, greater) effects on the WOMAC Index than other CIM interventions; e.g., acupuncture, leeches, and Tai Chi [38,39,40,41,42]. Direct comparisons with the results of this study are possible only to a limited extent, because other studies differed in part fundamentally from the present study in terms of study designs, intervention, and implementation.

This study had several strengths and limitations, such as the fact that the nature of the study did not allow for blinding of participants and therapists. These strengths and limitations are discussed in detail in the parent publication [3]. In the context of these secondary analyses, there was one minor methodological difference compared to the parent study. The multiple imputation was recalculated, and the 20 imputed datasets were compressed into a final single dataset, in contrast to the previous study; however, without any relevant changes. In this publication, the true predictive accuracy of the classifier C5.0 (Table S2 in the Supplementary Materials) could not be estimated because we did not have enough patients. In general, the predictive accuracy of a classifier such as C5.0 is estimated either by sampling (extracting a random sample from the patients’ file, constructing a classifier from the sample, and then testing the classifier on a disjoint collection of patients), or by using a separate validation file. However, this type of estimate is in general unreliable unless the numbers of patients used to build and evaluate the classifier are both sufficiently large (in our case, each file should have at least 100–150 patients).

## 5. Conclusions

These secondary analyses suggested that the therapeutic effects could not be explained by the therapies themselves alone, although they were the most important factors in the models. This can be explained against the background of numerous possible factors influencing the results of clinical studies such as this one. This is not a specific feature of CIM studies, but a generic aspect of clinical research, and more so of whole medical system (WMS) research. An awareness of this can be an essential tool for designing future clinical studies of complex CIM and Ayurveda interventions. Thus, within the framework of WMS research on Ayurveda (and other TMS), it may be possible in the future to further elaborate which specific individual factors are decisive for the success of such therapeutic approaches in order to systematically optimize study interventions and consecutively expand treatment options with elements from Ayurvedic medicine [15].

## Figures and Tables

**Table 1:** Estimated marginal means of changes in the WOMAC Index from baseline to 12 weeks and to 12 months, standard error, 95% CI, mean difference based on the marginal means and significance for the mean difference. Therapy, gender, and patients’ expectations for therapies received as fixed factors and WOMAC-A (pain), WOMAC-B (stiffness), and WOMAC-C (functional limitations), all three at baseline, as covariates.

	Therapy	Mean	Std. Error	95% Confidence Interval		
				Lower Bound	Upper Bound	Significance
WOMAC Index from baseline to 12 weeks	Ayurveda	46.7	6.7	33.5	59.9	
	Conventional therapy	26.4	6.7	13.2	39.7	
	Difference (Ayurveda—conventional therapy)	20.3	9.5	1.5	39.0	0.034
WOMAC Index from baseline to 12 months	Ayurveda	34.7	7.0	20.8	48.6	
	Conventional therapy	25.4	7.6	10.3	40.6	
	Difference (Ayurveda—conventional therapy)	9.3	10.4	−29.8	10.3	0.375

**Table 2:** Tests of between-subject effects with dependent variable: changes in WOMAC Index from baseline to 12 weeks and to 12 months. Therapy, gender, and patients’ expectations for therapy received were fixed factors; and WOMAC-A (pain), WOMAC-B (stiffness), and WOMAC-C (functional limitations), all three at baseline, were covariates.

	Model	F	df	Significance	Partial Eta Squared	Observed Power
12 weeks	Corrected model	8.09	19	<0.001	0.54	100.0%
	Therapy	7.43	1	0.007	0.054	77.2%
	Functional limitations at baseline	11	1	0.001	0.078	91.0%
12 months	Corrected model	4.63	19	<0.001	0.44	100.0%
	Therapy	1.9	1	0.171	0.017	27.7%
	Functional limitations at baseline	5.95	1	0.016	0.051	67.7%

**Table 3:** Significance, partial eta squared, and observed power for the independent variables in various generalized models (tests of between-subject effects). Therapy and functional limitations were the only significant predictors for the WOMAC Index from baseline to 12 weeks in all models with WOMAC subscales.

Tests of Between-Subject Effects							
	Dependent Variable: Changes in WOMAC Index from Baseline to 12 Weeks						
	Source	Type III Sum of Squares	df	F	Sig.	Partial Eta Squared	Observed Power
Model I	WOMAC Index baseline	108,356.6	1	104.0	<0.001	0.413	100.0%
	Therapy	37,834.5	1	36.3	<0.001	0.197	100.0%
Model II	WOMAC-A pain baseline	82,636.9	1	68.0	<0.001	0.315	100.0%
	Therapy	37,968.6	1	31.2	<0.001	0.171	100.0%
Model III	WOMAC-B stiffness baseline	67,526.4	1	51.2	<0.001	0.257	100.0%
	Therapy	34,093.8	1	25.9	<0.001	0.149	99.9%
Model IV	WOMAC-C functional limitations baseline	104,845.2	1	98.4	<0.001	0.399	100.0%
	Therapy	37,328.5	1	35.0	<0.001	0.191	100.0%
Model V	WOMAC-A pain baseline	2122.2	1	2.0	0.158	0.014	29.1%
	WOMAC-B stiffness baseline	1630.1	1	1.5	0.216	0.010	23.5%
	WOMAC-C functional limitations baseline	12,576.4	1	11.9	0.001	0.076	92.9%
	Therapy	37,786.1	1	35.8	<0.001	0.197	100.0%
Model VI	WOMAC-A pain baseline	616.4	1	0.6	0.430	0.006	12.3%
	WOMAC-B stiffness baseline	4302.8	1	4.4	0.039	0.041	54.6%
	WOMAC-C functional limitations baseline	11,001.1	1	11.2	0.001	0.097	91.3%
	Age	53.4	1	0.1	0.816	0.001	5.6%
	Duration of pains (years)	35.9	1	0.0	0.849	0.000	5.4%
	BMI	802.1	1	0.8	0.368	0.008	14.6%
	Therapy	14,222.4	1	14.5	<0.001	0.122	96.5%
	Gender	36.1	1	0.0	0.848	0.000	5.4%
	Expectations for Ayurveda	9572.7	4	2.4	0.051	0.086	68.2%
	Expectations for conventional therapy	2068.5	4	0.5	0.716	0.020	17.3%

## Data Availability

Not applicable.

## Supplementary Materials

The following supporting information can be downloaded at: https://www.mdpi.com/article/10.3390/jcm11113047/s1, Table S1: Baseline characteristics, Table S2: Classification of patients with “clinical improvement” (C5.0 model), Figure S1: Effect changes in the WOMAC Index from baseline to 12 weeks, Figure S2: Exhausted CHAID model for changes in the WOMAC Index from baseline to 12 weeks, including the factors of therapy and WOMAC subscale functional limitations at baseline.
