# Supplementary material for: Ayurveda in Knee Osteoarthritis—Secondary Analyses of a Randomized Controlled Trial
Source: J Clin Med. 2022 May 28;11(11):3047. doi: 10.3390/jcm11113047 (PMC9181350; doi:10.3390/jcm11113047)
Supplement: Supplementary file 1 [file jcm-11-03047-s001.zip › jcm-1723576-supplementary.pdf]

Supplementary Materials S1

Table S1: Baseline characteristics

Characteristic		All patients	Ayurveda	Conventional
		(n=151)	(n=77)	(n=74)
Mean age (SD), y		61.2 (6.6)	60.9 (6.5)	61.5 (6.6)
Mean Body mass index (SD), kg/m^2		26.1 (3.9)	25.8 (3.7)	26.4 (4.2)
> 10 years of school, n (%)		81 (54.4)	42 (56.0)	39 (52.7)
Mean duration of knee pain (SD), y		9.4 (8.1)	9.7 (9.1)	9.0 (7.0)
Patients with concomitant diagnoses (CD), n (%)		140 (92.7)	71 (92.2)	69 (93.2)
	Mean number of CD (SD)	4.3 (2.5)	4.4 (2.6)	4.1 (2.4)
	Patients with 1-2 CD, n (%)	27 (17.9)	13 (16.9)	14 (18.9)
	Patients with 3-4 CD, n (%)	48 (31.8)	22 (28.6)	26 (35.1)
	Patients with ≥ 5 CD, n (%)	65 (43.0)	36 (46.8)	29 (39.2)
Medication intake for knee OA		73 (48.3)	34 (44.2)	39 (52.7)
Mean systolic blood pressure (SD), mmHg		139.4 (16.8)	137.3 (16.1)	141.5 (17.3)
Mean diastolic blood pressure (SD), mmHg		85.6 (9.4)	84.1 (9.6)	87.1 (9.1)
Mean VAS score for knee pain (SD), mm		57.7 (11.7)	56.9 (11.7)	58.6 (11.7)
WOMAC, mean (SD)				
	Global scale	92.6 (42.2)	91.1 (40.3)	94.2 (44.4)
	Pain subscale	19.3 (8.5)	19.0 (8.1)	19.6 (9.0)
	Stiffness subscale	9.9 (4.7)	9.8 (4.7)	10.1 (4.7)
	Function subscale	63.4 (31.8)	62.3 (30.6)	64.5 (33.1)
PDI, mean (SD)		23.8 (11.4)	22.6 (10.6)	25.1 (12.1)
PDI, mean (SD)		23.8 (11.4)	22.6 (10.6)	25.1 (12.1)
SES, mean (SD)				
	Affective	27.1 (8.2)	27.3 (8.8)	26.9 (7.6)
	Sensory	18.2 (5.7)	18.3 (5.6)	18.1 (5.8)
POMS, mean (SD)				
	Depression factor	1.5 (0.9)	1.5 (1.0)	1.4 (0.9)
	Fatigue factor	1.8 (0.9)	1.8 (0.9)	1.8 (0.9)
	Vigor factor	2.0 (0.7)	2.0 (0.6)	2.0 (0.7)
	Anger factor	1.7 (0.9)	1.8 (0.9)	1.7 (0.8)
SF-36, mean (SD)				
	Physical component summary	33.2 (7.7)	33.4 (7.4)	33.0 (8.1)
	Mental component summary	51.3 (11.3)	50.4 (12.1)	52.3 (10.5)
NRS (11-point 0-10), mean (SD)				
	Pain at rest	3.4 (2.3)	3.4 (2.3)	3.4 (2.3)
	Pain during movement	5.6 (1.9)	5.4 (2.0)	5.9 (1.7)

	Everyday bothersomeness through pain	5.3 (2.0)	5.1 (2.1)	5.6 (1.9)
	Sleep quality	5.6 (2.5)	5.2 (2.5)	6.0 (2.5)
Likert scales (7-point, 0-6), mean (SD)				
Patient´s expectations of Ayurveda therapy				
	Reduction of OA complaints	4.8 (1.1)	4.8 (1.1)	4.8 (1.0)
	Overall effectiveness	4.7 (1.2)	4.6 (1.2)	4.9 (1.1)
	Comprehensibility	4.6 (1.3)	4.6 (1.3)	4.6 (1.3)
Patient´s expectations of conventional therapy				
	Reduction of OA complaints	3.8 (1.3)	3.7 (1.3)	3.9 (1.4)
	Overall effectiveness	3.7 (1.2)	3.4 (1.1)	4.0 (1.2)
	Comprehensibility	4.1 (1.4)	4.0 (1.3)	4.2 (1.4)
Physician´s expectations of Ayurveda therapy				
	Reduction of OA complaints	5.0 (1.0)	5.1 (1.0)	5.0 (1.0)
	Overall effectiveness	4.5 (0.9)	4.5 (1.0)	4.5 (0.9)
	Comprehensibility	4.7 (1.1)	4.7 (1.1)	4.7 (1.0)
Physician´s expectations of conventional therapy				
	Reduction of OA complaints	3.5 (0.9)	3.4 (1.0)	3.5 (0.8)
	Overall effectiveness	3.0 (0.9)	3.0 (0.9)	3.0 (0.8)
	Comprehensibility	3.8 (1.1)	3.7 (1.1)	3.8 (1.1)
Study center, n (%)				
Study center 1		121 (80,1)	61 (40,4)	60 (39,7)
Study center 2		30 (19,9)	16 (10,6)	14 (9,3)

Table S2: Classification of patients with "clinical improvement" (C5.0 model)

Rule 1:
Therapy = Ayurveda
WOMAC-C baseline > 6
→ Clinical improvement
Rule 2: Therapy = Conventional
WOMAC-B stiffness baseline > 10
→ Clinical improvement
Rule 3:
Therapy = Ayurveda
WOMAC-C baseline ≤ 6
→ Clinical improvement
Rule 4:
Therapy = Conventional
WOMAC-B stiffness baseline ≤ 10
Gender = Male
→ No improvement or worsening
Rule 5:
Therapy = Conventional therapy
WOMAC-B stiffness baseline ≤ 10
Gender = Female
Patients' expectations of the therapy received= medium to high
WOMAC-A pain baseline ≤ 11
→ Clinical improvement
Rule 6:
Therapy = Conventional therapy
WOMAC-B stiffness baseline ≤ 10
Gender = Female
Patients' expectations of the therapy received= medium to high
WOMAC-A pain baseline > 11
→ No clinical improvement or worsening
Rule 7:
Therapy = Conventional therapy
WOMAC-B stiffness baseline ≤ 10
Gender = Female
Patients' expectations of the therapy received= low
duration of knee pain (years) > 24
→ No clinical improvement or worsening
Rule 8:
Therapy = Conventional therapy
WOMAC-B stiffness baseline ≤ 10
Gender = Female
Patients' expectations of the therapy received= low
duration of knee pain (years) ≤ 24
WOMAC-B stiffness baseline > 2
→ Clinical improvement
Rule 9:
Therapy = Conventional therapy
WOMAC-B stiffness baseline ≤ 10
Gender = Female
Patients' expectations of the therapy received= low
duration of knee pain (years) ≤ 24
WOMAC-B stiffness baseline ≤ 2
→ No clinical improvement or worsening
Default class: Clinical improvement

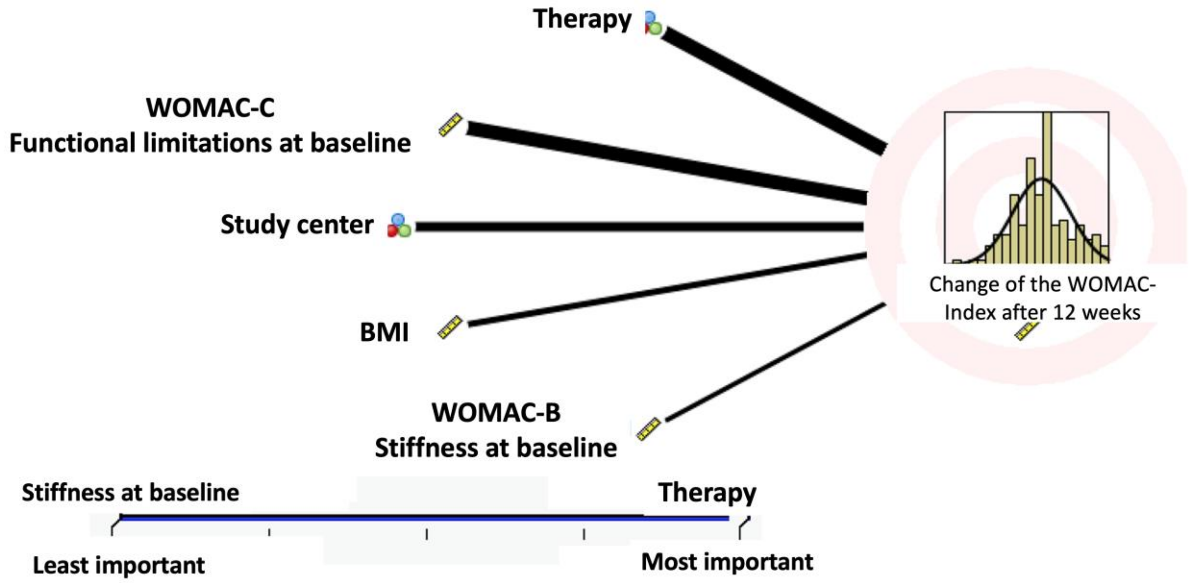

Figure S1: Effect changes of the WOMAC-Index from baseline to 12 weeks. Therapy and functional limitations are the most important predictors for the outcome in a regression model.

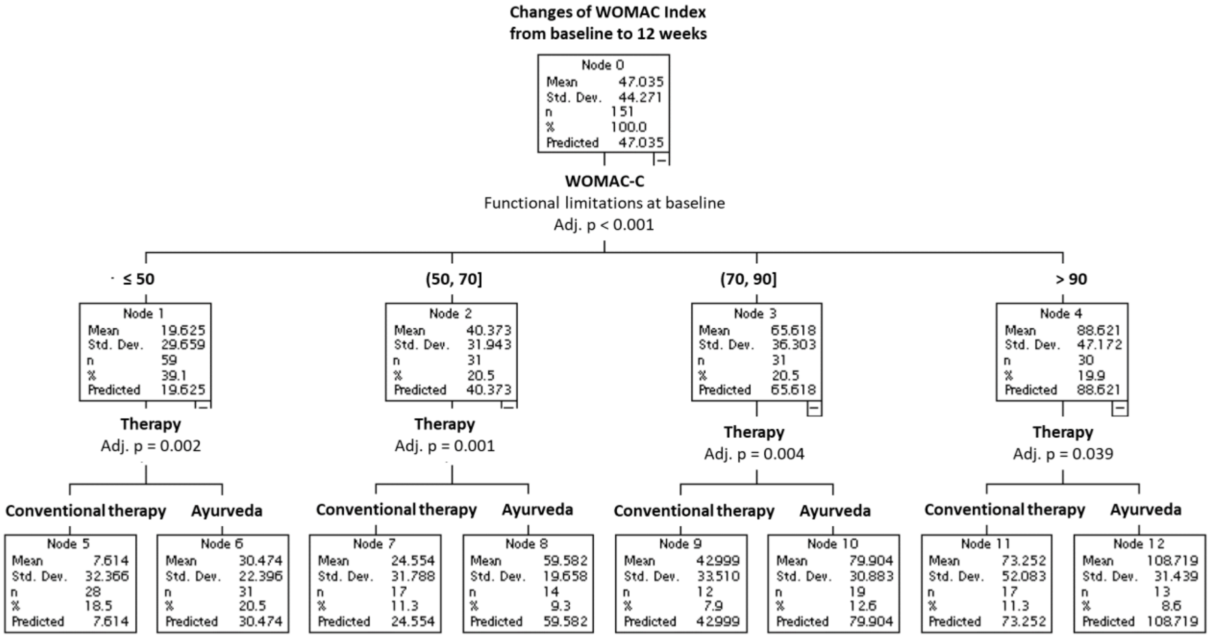

Figure S2: Exhausted CHAID model for changes of WOMAC-Index from baseline to 12 weeks including the factors therapy and WOMAC subscale functional limitations at baseline.
